# PTPσ Knockdown in Lampreys Impairs Reticulospinal Axon Regeneration and Neuronal Survival After Spinal Cord Injury

**DOI:** 10.3389/fncel.2020.00061

**Published:** 2020-03-19

**Authors:** William Rodemer, Guixin Zhang, Isabelle Sinitsa, Jianli Hu, Li-qing Jin, Shuxin Li, Michael E. Selzer

**Affiliations:** ^1^Shriners Hospitals Pediatric Research Center, Lewis Katz School of Medicine, Temple University, Philadelphia, PA, United States; ^2^College of Science and Technology, Temple University, Philadelphia, PA, United States; ^3^Department of Anatomy and Cell Biology, Lewis Katz School of Medicine, Temple University, Philadelphia, PA, United States; ^4^Department of Neurology, Lewis Katz School of Medicine, Temple University, Philadelphia, PA, United States

**Keywords:** spinal cord injury, CPSGS, PTPRS, lampreys, cell death, regeneration

## Abstract

Traumatic spinal cord injury (SCI) results in persistent functional deficits due to the lack of axon regeneration within the mammalian CNS. After SCI, chondroitin sulfate proteoglycans (CSPGs) inhibit axon regrowth *via* putative interactions with the LAR-family protein tyrosine phosphatases, PTPσ and LAR, localized on the injured axon tips. Unlike mammals, the sea lamprey, *Petromyzon marinus*, robustly recovers locomotion after complete spinal cord transection (TX). Behavioral recovery is accompanied by heterogeneous yet predictable anatomical regeneration of the lamprey’s reticulospinal (RS) system. The identified RS neurons can be categorized as “good” or “bad” regenerators based on the likelihood that their axons will regenerate. Those neurons that fail to regenerate their axons undergo a delayed form of caspase-mediated cell death. Previously, this lab reported that lamprey PTPσ mRNA is selectively expressed in “bad regenerator” RS neurons, preceding SCI-induced caspase activation. Consequently, we hypothesized that PTPσ deletion would reduce retrograde cell death and promote axon regeneration. Using antisense morpholino oligomers (MOs), we knocked down PTPσ expression after TX and assessed the effects on axon regeneration, caspase activation, intracellular signaling, and behavioral recovery. Unexpectedly, PTPσ knockdown significantly impaired RS axon regeneration at 10 weeks post-TX, primarily due to reduced long-term neuron survival. Interestingly, cell loss was not preceded by an increase in caspase or p53 activation. Behavioral recovery was largely unaffected, although PTPσ knockdowns showed mild deficits in the recovery of swimming distance and latency to immobility during open field swim assays. Although the mechanism underlying the cell death following TX and PTPσ knockdown remains unknown, this study suggests that PTPσ is not a net negative regulator of long tract axon regeneration in lampreys.

## Introduction

Spinal cord injury (SCI) results in persistent functional deficits in mammals because injured CNS axons fail to regenerate. This failure results from both inhibitory factors in the extracellular environment and intrinsic limitations to regenerative capacity (Tran et al., 2018). Among the most potent and well-described environmental factors are the chondroitin sulfate proteoglycans (CSPGs), which are abundantly expressed around CNS lesion as a key component of the glial scar (Stichel et al., 1999). Digesting CSPGs with chondroitinase increases axon outgrowth on a CSPG substrate* in vitro*, promotes sprouting of spared fibers *in vivo* and enhances functional recovery from SCI in animal models (Zuo et al., 1998; Bradbury et al., 2002; Barritt et al., 2006; Houle et al., 2006).

The LAR-family of receptor-type protein tyrosine phosphatases (RPTPs), including LAR and PTPσ, have been identified as putative CSPG receptors in mammals (Shen et al., 2009; Fisher et al., 2011). Consisting of approximately 1.9 × 10^3^ amino acids for their longest splice variants, these transmembrane receptors are structurally highly similar (Chagnon et al., 2004). Starting from the extracellular N-terminal, they contain three Ig-like domains, the first of which interacts with CSPGs *via* conserved lysine residues. These domains are followed by a number of fibronectin type III (FNIII) repeats, which vary depending on splicing. Intracellularly, there are two phosphatase domains, D1 and D2. D1 is generally believed to be active while D2 is considered structurally inactive and may serve in a more regulatory role. Genetic deletion or peptide inhibition of LAR and PTPσ has been reported to enhance CNS axon regeneration after SCI, likely *via* RhoA-dependent mechanisms (Shen et al., 2009; Fisher et al., 2011; Lang et al., 2015; Ohtake et al., 2016). Notably, LAR and PTPσ often colocalize within the same neuronal populations. In mammalian studies, targeting LAR or PTPσ in isolation was sufficient to elicit pro-regenerative functional effects, but inhibiting both receptors simultaneously additively enhanced axon outgrowth *in vitro* (Ohtake et al., 2016).

Nevertheless, the role of LAR-family RPTPs in long tract regeneration remains unclear. Although, PTPσ knockdown has been reported to enhance corticospinal tract axon regeneration, the use of partial injury models and the complexity of the mammalian CNS make it difficult to distinguish true regeneration from compensatory sprouting of spared fibers (Fry et al., 2010). Moreover, the effects of receptor inhibition on axon regeneration appear to be context-dependent. Following sciatic nerve crush, an early report found PTPσ deletion enhanced sensory axon regeneration but increased pathfinding errors (McLean et al., 2002). However, other studies using sciatic nerve injury models reported that deletion of LAR or PTPδ, a third LAR-family RPTP member, reduced sensory axon regeneration and worsened outcomes (Xie et al., 2001; Van der Zee et al., 2003). As a further complication, RPTPs have been reported to modulate the immune responses to CNS injury, as well as the survival and differentiation of local oligodendrocyte precursors and neural progenitors (Dyck et al., 2015, 2018, 2019; Ohtake et al., 2017).

Unlike mammals, sea lampreys (*Petromyzon marinus*) recover robustly from complete spinal cord transection (TX) despite the presence of a CSPG-rich scar that forms around the TX site. In lampreys, peak CSPG expression occurs approximately 2 weeks after TX and then slowly returns to normal levels by 10 weeks post-TX (Lurie et al., 1994; Zhang et al., 2014). Behavioral recovery is accompanied by true regeneration of reticulospinal (RS) axons, which facilitate descending control of locomotion. The RS system comprises approximately 2,000 neurons in total that are distributed within the hindbrain and posterior aspect of the midbrain. Of these, there are 18 pairs of large identified neurons, which project to at least the level of the cloaca. Following complete TX, all descending connections are severed. However, after a period of retraction, RS axons begin to regenerate, reaching the TX scar by approximately 3 weeks (Yin and Selzer, 1983). This regeneration is heterogeneous but predictable. Approximately half of the identified neurons will reliably regenerate to at least 5 mm beyond the TX, while the other half fail to regenerate and undergo a severely delayed form of apoptosis that peaks between 12 and 16 weeks post-TX (Shifman et al., 2008). As the regeneration probability for each identified neuron is broadly stable across studies, they can be semi-permanently classified as “good” or “bad” regenerators (Table 1). However, the exact definitions can vary depending on the study. In this report, neurons with regeneration probabilities >50% were considered “good” regenerators.

**Table 1:** Regeneration probabilities for the identified reticulospinal (RS) neurons.

	Good regenerators ( >50% Regen. Probability)									
Neuron	I6	I4	B6	M4	Mth’	I5	I3	B5		
Regen. Prob. (%)	57	59	61	63	69	69	70	70		
	**Bad regenerators (<50% Regen. Probability)**									
Neuron	I1	B3	Mth	B4	M2	M3	B1	I2	B2	M1
Regen. Prob. (%)	4	6	7	11	13	13	19	37	46	48

Recently, this lab reported that the CSPG-receptor, PTPσ, is expressed preferentially in bad regenerators, both before and after TX (Zhang et al., 2014). Consequently, we hypothesized that knocking down PTPσ with antisense morpholino oligomers (MOs) would promote regeneration and reduce retrograde neuronal death after TX.

## Materials and Methods

### Animals and Surgery

Wild-caught larval sea lampreys, approximately 8–12 cm in length (3–5 years old), obtained from the streams of Lake Michigan were maintained in freshwater tanks at room temperature until use. All animal procedures described in this report were performed with approval from Temple University’s Institutional Animal Care and Use Committee. The total number of animals used is summarized in Table 2.

**Table 2:** Summary of animal experiments.

Experiment	Sample size (*N*)													
	MO	Weeks post-TX												
		−1	1	2	3	4	5	6	7	8	9	10	20	24
MO efficiency	Ctrl	.	.	.	.	9^a^	.	.	.	.	.	.	.	3
	PTPσ	.	.	.	.	8^a^	.	.	.	.	.	.	.	2
*In situ* hybridization	Ctrl	.	.	7	.	.	.	.	.	.	.	.	.	.
	PTPσ	.	.	8	.	.	.	.	.	.	.	.	.	.
RS axon regeneration	Ctrl	.	.	.	.	.	.	.	.	.	.	9	.	.
	PTPσ	.	.	.	.	.	.	.	.	.	.	11	.	.
FLICA	Ctrl	.	.	5	.	9	.	.	6	.	.	6	6	.
	PTPσ	.	.	6	.	8	.	.	6	.	.	6	6	.
Swimming behavior assay^b^	Naïve	58	.	.	.	.	.	.	.	.	.	.	.	.
	Ctrl	.	17	17	5	15	15	15	15	15	15	10	.	.
	PTPσ	.	17	17	5	15	15	15	15	14	14	8	.	.
Western blot, brain	Ctrl	.	.	4	.	.	.	.	.	.	.	.	.	.
	PTPσ	.	.	4	.	.	.	.	.	.	.	.	.	.
Western blot, spinal cord	Ctrl	.	.	5	.	.	.	.	.	.	.	.	.	.
	PTPσ	.	.	4	.	.	.	.	.	.	.	.	.	.
Immunofluorescence/Histochemistry	Ctrl	.	.	4	.	.	.	.	.	.	.	.	.	.
	PTPσ	.	.	3	.	.	.	.	.	.	.	.	.	.

*^a^Transfection efficiency was assessed using the FLICA 4 week post-TX cohort. ^b^Animal sample size represents animals followed longitudinally before the injury and from 4 to 10 weeks or 1 to 10 weeks post-TX*.

Lampreys were anesthetized by immersion in 0.1% tricaine methanesulfonate (MS-222, Western Chemical Inc., Washington, DC, USA) in Ringer’s solution and the spinal cords were exposed by a small longitudinal incision along the dorsal midline in the gill region. Spinal cords were completely transected with Castroviejo scissors at the level of the 5th gill. The completeness of the TX was confirmed by visual inspection of the severed spinal cord stumps. To retrogradely transfect RS neurons, a small gel foam pledget soaked with 1 μl of 1 mM fluorescein-tagged MOs was gently inserted on top of the severed spinal cord immediately after the injury. RS neurons that were successfully transfected could then be identified by their fluorescent labeling. Afterward, the animals were allowed to recover on ice for 2 h; then the gel foam was removed and the animals placed in cold water tanks overnight. The following day, animals were returned to room temperature tanks until needed.

To evaluate true axon regeneration beyond the lesion, some lampreys received a second complete TX, 5 mm caudal to the first, at 10 weeks post-(1st) transfection. Regenerated RS neurons were retrogradely labeled by applying a gel foam pledget soaked in 1 μl of a solution of 5% dextran tetramethylrhodamine (DTMR, 10,000 MW; Em. 550 nm, Ex. 580 nm; Invitrogen, D1817) in Tris-HCl buffer (pH 7.4) to the lesion. Animals were euthanized 1 week later by immersion in saturated benzocaine and brains were dissected, fixed in 4% paraformaldehyde, and imaged under widefield fluorescent (Nikon 80i, 4× objective) or confocal (Nikon C2, 10× objective) microscopy. Morphant identified neurons were assessed individually based on position and morphology. A neuron was counted as “regenerated” if the cell was double positive for fluorescein-conjugated MO and dextran dye. Statistical analysis was performed with GraphPad Prism 8 using a two-tailed student’s *t*-test.

To assess TX-induced caspase activation, lampreys were euthanized at 2, 4, 7, 10, or 20 weeks post-TX and processed for *fluorochrome-labeled inhibitors of caspase activation* (FLICA) as previously described (Barreiro-Iglesias and Shifman, 2012; Hu et al., 2013). Briefly, freshly dissected brains were immersed in sulforhodamine poly caspase FLICA reagent (SR-VAD-FMK; Ex. 570 nm, Em. 590 nm; Image-IT Live Red Poly Caspases Detection Kit, Cat I305101, Thermo Fisher Scientific, Waltham, MA, USA), which irreversibly binds activated caspases 1, 3, 4, 5, 6, 7, 8, and 9, then thoroughly washed, flat-mounted and fixed with 4% paraformaldehyde. Brains were imaged by wide-field fluorescence microscopy (Nikon 80i, 4× objective). Morphant- and FLICA-positive identified neurons were counted, and the results were analyzed with GraphPad Prism 8 using one-way ANOVA with Sidak *post hoc* multiple comparisons tests.

### Antisense Morpholino Oligomers

Lamprey LAR-family RPTPs are not annotated in the lamprey reference genome (*P.marinus_7*.0). However, by aligning PTPσ DNA sequences from mammals to lampreys using basic local alignment search tools (BLAST), genome regions encoding PTPσ were identified and partially cloned from cDNA (Zhang et al., 2014). Introns were identified by comparing genomic sequences with the cDNA *exonic* sequence. Using Gene Tools LLC custom oligomer design service, we designed a *splice acceptor* and *splice donor* pair of MOs complementary to the exon-intron junctions flanking an exon encoding a region near the 3rd Ig-like domain of lamprey PTPσ (${\text{MO}}_{{\text{PTP}}_{\text{σ}}}$; GL479748:15741-15933; splice acceptor MO sequence, 5′-AGA GCC TGC GGC AAG TCA GGC AGA A-3′; splice donor MO sequence, 5′-CTG CTG CTG CGT ATC CTC ACC CTT C-3′). For optimal efficiency, a 1:1 suspension of splice acceptor and splice donor oligomers were used for all experiments (approximately 0.5 mM each suspended in water). The Gene Tools LLC Standard Control MO (MO_Ctrl_) was selected for the negative control (MO sequence, 5′-CCT CTT ACC TCA GTT ACA ATT TAT A-3′). This MO targets a rare mutation at position 705 in human beta-globin pre-mRNA and is recommended by Gene Tools LLC for having minimal biological activity. All MOs used in this study were covalently bound at the 3′ end to fluorescein (Ex 501.5 nm, Em 524.5 nm).

### Open Field Behavioral Assay

Naïve and TX’d lamprey locomotion was assessed 3 days per week, beginning 1 week before injury until 10 weeks post-injury. Individual animals were gently placed in a Nalgene pan shallowly filled with aquarium water and monitored by an overhead camera for 2 min of free swim. Swimming quality was assessed on a semiquantitative “movement” score: (0) no movement below lesion; (1) spastic movements below lesion; (2) brief forward movements; (3) persistent abnormal swimming, <80% of time with dorsal side up; (4) persistent abnormal swimming, >80% of time dorsal side up; and (5) normal swimming, 100% of time dorsal side up. Videos were processed with ANY-maze animal tracking software (Stoelting) to quantify swimming parameters including total distance traveled, maximum swimming speed, time spent mobile, and latency to immobility. Statistical analysis of weekly averages was performed with Graphpad Prism 8 using two-way ANOVA with a *post hoc* Sidak multiple comparisons test.

### Western Blot

To determine protein expression levels, freshly dissected lamprey brains or spinal cords (from the 2nd gill to 5 mm distal to the TX) were snap-frozen in liquid nitrogen and stored at −80°C until use. Frozen lamprey specimens were thawed on ice and suspended in cold lysis buffer (CelLytic MT; Sigma–Aldrich, St. Louis, MO, USA, Cat C3228) supplemented with a cocktail of protease and phosphatase inhibitors (Halt Protease and Phosphatase Inhibitor Cocktail 100×, Thermo Fisher Scientific, Waltham, MA, USA) and homogenized by sonication. Supernatants were collected following brief centrifugation to remove debris and protein concentration was assessed by Bradford Assay (Bio-Rad, Cat 500-0006). The samples, containing 25 μg of protein supplemented with reducing reagent (Invitrogen, Cat NP0004) and loading buffer (Invitrogen, NP0007), were denatured by heating at 75°C. Denatured samples were loaded into a 4–12% NuPage Bis-Tris mini-gel (Invitrogen, Cat NP0321BOX), separated by electrophoresis, then wet-transferred onto a PVDF membrane.

After transfer, PVDF membranes were washed thoroughly with TBS, blocked with 5% non-fat milk, and incubated with primary antibody diluted in 1% non-fat milk overnight at 4°C. The following day, membranes were washed thoroughly with TBS, and incubated with near-IR secondary antibodies in PBS Blocking Buffer (Li-Cor, Cat 927-40000) with 0.1% Tween-20 and 0.01% SDS final concentrations. Membranes were imaged with an Odyssey CLx Imaging System (Li-Cor). To assess additional proteins, the immunoblotting procedure was sequentially repeated.

Signal intensity was measured and blots were processed for presentation using Image Studio Lite (version 4.0, Li-Cor). Statistical analysis was performed on signal intensity values with GraphPad Prism 8 using two-tailed student’s *t*-tests. Primary antibodies used in this study include: rabbit anti-phospho-Akt mAB (T308; 1:1,000; Cell Signaling Technology, Danvers, MA, USA, Cat 2965; RRID:AB_2255933), rabbit anti-pan-Akt mAB (1:3,000; Cell Signaling Technology, Danvers, MA, USA, Cat 4691; RRID:AB_915783), rabbit anti-p53 (1:1,000; AbClonal, Cat A5761; RRID:AB_2766515) and mouse anti-Actin mAB (1:10,000; Millipore, Kankakee, IL, USA, Cat 1501; RRID:AB_2223041). Primary antibodies were detected with IRDye 680 or IRDye 800 secondary antibodies (1:20,000, Li-Cor).

### Immunofluorescence

Flat-mounted, PFA fixed, lamprey brains were dehydrated, embedded in paraffin, horizontally sectioned on a microtome (10 μm section thickness), and mounted on ColorFrost Plus slides. The samples were then processed for immunofluorescence as follows: rehydrated to PBS; incubated with Citrate Buffer (pH 6.0) for antigen retrieval (20 min boiling then 20 min RT); blocked with PBS with 0.1% bovine serum albumin and 0.2% Triton X-100 for 1 h RT; incubated with rabbit anti-p53 primary antibody diluted in blocking buffer overnight at 4°C (1:200; AbClonal, Cat A5761; RRID:AB_2766515); incubated with anti-rabbit AlexFluor+ 594 secondary antibody for 1 h RT in the dark (1:400; Invitrogen, Cat A32740; RRID:AB_2762824); and mounted under glass coverslips with Fluoromount-G (Southern Biotech, Birmingham, AL, USA). The samples were thoroughly washed with PBS between each step. Fluorescence images were captured *via* confocal microscopy (Nikon C2 microscope, 10× objective) and processed with NIS-Elements and Adobe Photoshop.

### Microglia Staining

To assess the effects of PTPσ knockdown on the local immune response to SCI, at 2 weeks pTX, microglia were stained by isolectin B4 –HRP (1:200; Sigma–Aldrich, St. Louis, MO, USA, Cat# L5391) and DAB histochemistry was performed on 10 μm horizontal sections (i.e., cut parallel to the dorsal surface) of fixed, paraffin-embedded lamprey spinal cord. For each spinal cord, the number of labeled microglia were recorded from each of four serial sections proximal to the injury, near the median plane. Macrophages or microglia residing within the central canal were excluded. The region assessed included the TX site and extended 600 μm Rostralward. Statistical analysis was performed on the mean number of microglia observed per horizontal section (for MO, *n* = 2 animals × 4 sections = 8; for MO_Ctrl_, *n* = 3 animals × 4 sections = 12) using a two-tailed student’s *t-test*.

### *In situ* Hybridization

Free-floating, fixed wholemount lamprey brains were imaged by widefield fluorescence microscopy (Nikon 80i), then processed for ISH with a custom lamprey PTPσ RNA probe as previously described (Swain et al., 1994; Zhang et al., 2014). Briefly, brains were washed with PBS containing 0.1% Tween-20 and processed as follows: (1) immersed in hybridization solution (50% Formamide, 5× SSC, 100 μg/μl Torula-Yeast RNA, 100 μg/μl Wheat germ tRNA, 50 μg/μl Heparin, 0.1% Tween-20; 1 h at 50–55°C); (2) incubated with biotinylated PTPσ riboprobe (1.5 μg/ml) in hybridization solution (overnight at 50–55°C); (3) washed with hybridization solution (4× for 30 min at 50–55°C); (4) washed with a 1:1 mix of hybridization solution and PBS with 0.1% Tween-20 (2× for 15 min at 50–55°C); (5) washed with PBS containing 0.1% Tween-20 (2× for 30 min); (6) incubated in blocking solution (PBS containing 0.1% BSA Fraction V and 0.2% Triton X-100; 4× for 30 min); (7) incubated with streptavidin-AP (1:1000; Roche, Cat 11093266910) in blocking solution (overnight at 4°C); (8) washed with blocking solution (4× for 30 min); (9) washed with SMT buffer (100 mM NaCl, 50 mM MgCl_2_, 100 mM Tris pH 9.5, 0.1% Tween-20; 4× for 30 min); (10) incubated with ice-cold chromogen (NBT/BCIP, Roche, Cat 11681451001; 10 min) in SMT buffer; (11) washed with PBS (3× for 5 min); and (12) mounted on glass slides with PBS and imaged under reflected light stereomicroscopy.

Fluorescent and brightfield images were superimposed in Adobe Photoshop. Since, the images were taken under different conditions, cells did not perfectly overlap but could still be identified from position and morphology. Single and double-labeled cells were counted and the resulting values were analyzed statistically with GraphPad Prism 8. To provide a complete overview of PTPσ expression after TX with MO_Ctrl_ or ${\text{MO}}_{{\text{PTP}}_{\text{σ}}}$ treatment, a contingency analysis on all counted cells was performed using Fisher’s exact test [*α* = 0.05; categories: ${\text{MO}}_{{\text{PTP}}_{\text{σ}}}$-treated, PTPσ(+); ${\text{MO}}_{{\text{PTP}}_{\text{σ}}}$-treated, PTPσ(−); MO_Ctrl_-treated, PTPσ(+); and MO_Ctrl_-treated, PTPσ(−)]. As a complementary analysis, the mean number of PTPσ positive neurons per lamprey with MO_Ctrl_ or ${\text{MO}}_{{\text{PTP}}_{\text{σ}}}$ treatment was assessed with a two-tailed student’s *t*-test.

## Results

### MOs Reduced PTPσ Expression Among RS Neurons

Antisense MOs, which bind complementary target RNA *via* Watson-Crick base paring, have proven to be reliable vectors for inducing protein knockdown in lampreys (Zhang et al., 2015; Hu et al., 2017; Sobrido-Cameán et al., 2019). Importantly, MOs applied to the spinal cord stumps after TX are readily taken up by severed axons and retrogradely transported to their cell bodies in the brain (Zhang et al., 2015). MOs targeting lamprey PTPσ (MO) were designed to interact with intron-exon junctions flanking an exon encoding a region in the 3rd Ig-like domain towards the N-terminal. This was predicted to induce an exon skipping event to reveal a premature stop codon, at which point the transcript would be degraded *via* nonsense-mediated decay mechanisms.

Although the half-life of PTPσ in lampreys is unknown, the half-life of human PTPσ is estimated to be between 16 h and 2 days (Cohen et al., 2013; Xiao and Wu, 2017). Thus, PTPσ must be continually synthesized to maintain expression levels. Previous studies demonstrated that MOs applied to the cut ends of lamprey RS axons immediately after TX reach their perikarya in the brain within 1 week of injury and are effective in reducing protein expression of their target RNAs (Zhang et al., 2015; Hu et al., 2017). After an initial 10-day period of retraction, axons of the identified RS neurons begin to regenerate, reaching the edge of the CSPG-rich scar by 3 weeks post-TX (Yin and Selzer, 1983). Therefore, it is likely that inhibiting the translation of PTPσ will reduce its protein expression in axon tips at time points relevant to regeneration (Lurie et al., 1994).

**Figure 1:**
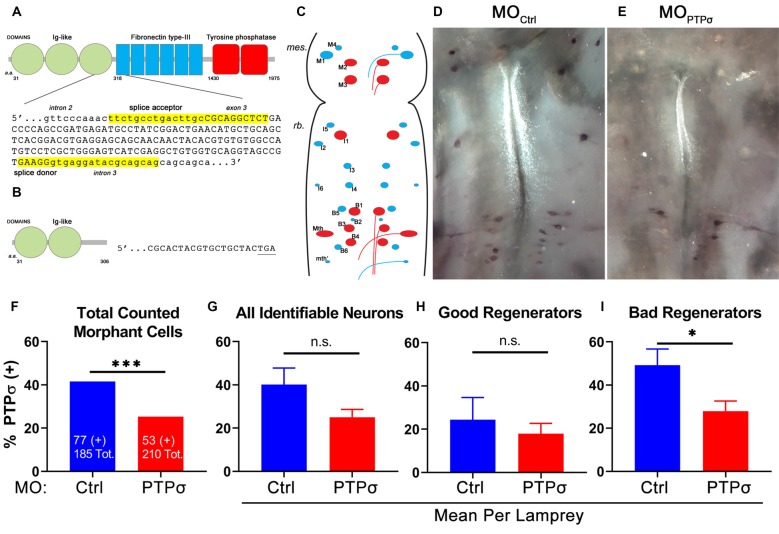
Splice-blocking morpholino oligomers (MOs) reduce PTPσ mRNA expression at 2 weeks post-transection (TX). **(A)** Schematic of wild type lamprey PTPσ with the genomic sequence targeted by the MOs. **(B)** Schematic of morphant PTPσ showing the premature stop codon produced by exon skipping. **(C)** Schematic of flat-mounted larval lamprey brain showing the identified RS neurons (“good regenerators,” blue; “bad regenerators,” red).** (D,E)** Representative micrographs of wholemount brain *in situ* hybridization using a PTPσ RNA probe at 2 weeks post-TX. **(F)** Contingency analysis with Fisher’s Exact Test on all counted cells. Significantly fewer RS neurons treated with ${\text{MO}}_{{\text{PTP}}_{\text{σ}}}$ were positive for PTPσ mRNA than controls. **(G–I)** Analysis with student’s *t*-tests revealed that significantly fewer bad regenerators treated with ${\text{MO}}_{{\text{PTP}}_{\text{σ}}}$ (*n* = 8) were positive for PTPσ than were those treated with MO_Ctrl_ (*n* = 7). Error bars show SEM. *p < 0.05; ***p < 0.001; n.s., non-significant.

To analyze PTPσ mRNA expression at the level of individual RS neurons, 2 weeks after TX, we performed *in situ* hybridization in wholemount lamprey brains, using biotinylated riboprobes designed to hybridize to the fibronectin-III domain of lamprey PTPσ (Figure 1). This region was chosen for specificity because it has low homology to the closely related LAR-family RPTPs, LAR and PTPδ (Zhang et al., 2014). Contingency analysis with Fisher’s exact test, using the total counts of identified neurons, demonstrated that ${\text{MO}}_{{\text{PTP}}_{\text{σ}}}$ lampreys had significantly fewer PTPσ positive RS neurons compared to MO_Ctrl_ (***p = 0.0006). Reductions in PTPσ expression were most evident among “bad regenerator” identified RS neurons, where PTPσ is most highly expressed. ${\text{MO}}_{{\text{PTP}}_{\text{σ}}}$ reduced the number of PTPσ-expressing “bad regenerator” RS neurons per lamprey by approximately 43% (*p = 0.03). Non-significant reductions in PTPσ were observed among “good regenerator” identified RS neurons (25% reduction, *p* = 0.25). Although knockdown was not complete, these results demonstrated that MO reduced PTPσ expression as designed.

### PTPσ Knockdown Impaired Axon Regeneration

**Figure 2:**
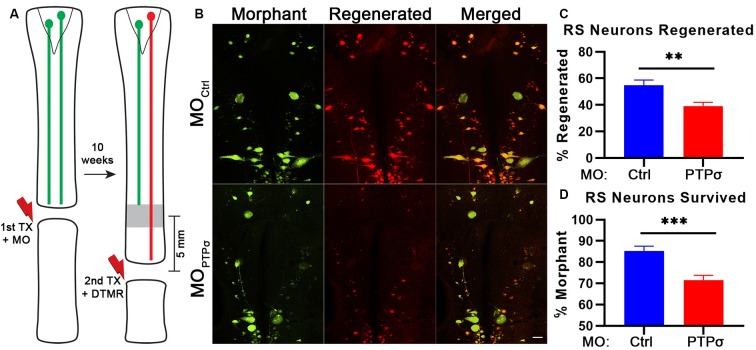
PTPσ knockdown impaired axon regeneration and long-term neuron survival. **(A)** Schematic of retrograde labeling procedure. At 10 weeks after TX and MO application, lampreys were subjected to a second TX, 5 mm caudal to the first, and regenerated axons were back labeled with DTMR. **(B)** Representative whole mounted brain maximum intensity projection confocal micrographs of MO-Ctrl or ${\text{MO}}_{{\text{PTP}}_{\text{σ}}}$ (green) and DTMR (red) labeled RS neurons (*n* = 10–12 animals). Scale, 100 μm.** (C–D)** Quantification of identified RS neuron regeneration (DTMR) and survival (MO-fluorescein). Statistical analysis performed with two-tailed student’s *t*-tests. Error bars show SEM. **p < 0.01; ***p < 0.001.

To assess axon regeneration following TX and PTPσ knockdown, we utilized a double-labeling strategy. Ten weeks after the initial TX, a second TX was made 5 mm caudal to the first and a fluorescent dextran tracer was applied to retrogradely label neurons with regenerated axons (Figure 2). Using this approach, unexpectedly, we observed significantly fewer regenerated identified RS neurons per lamprey following PTPσ knockdown relative to controls (~29% fewer regenerated neurons, **p < 0.01; Figure 2C). Interestingly, this impaired regeneration appeared to be largely due to reduced long-term neuron survival beginning around 10 weeks post-injury and worsening over time. By 20 weeks post-TX, 35% fewer identified RS neurons survived following PTPσ knockdown relative to controls (***p < 0.001; Figure 4B).

**Figure 3:**
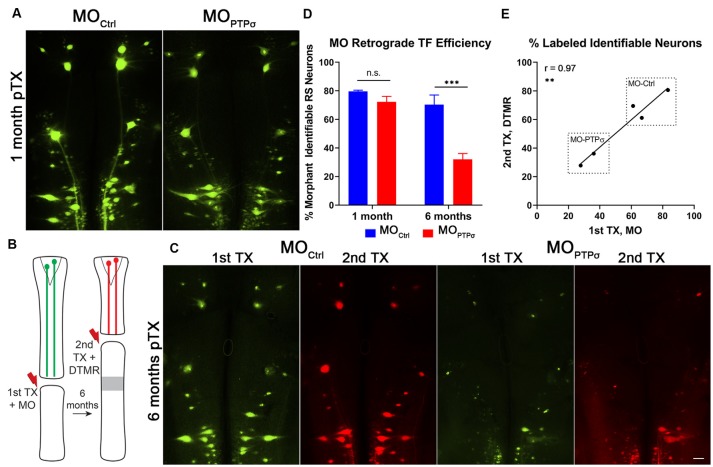
Morpholino labeling is a persistent proxy for cell survival. **(A)** Representative micrographs of fluorescent-MO labeling among RS neurons, 1 month after TX and MO application. **(B)** Experiment schematic for labeling surviving RS neurons. Six months after TX and transfection with ${\text{MO}}_{{\text{PTP}}_{\text{σ}}}$ (*n* = 2) or MO_Ctrl_ (*n* = 3), a second TX was made rostral to the first, close to the brain, and surviving RS neurons were back labeled with DTMR. **(C)** Representative micrographs of whole mounted lamprey brains showing MO transfected RS neurons (1st TX) and surviving RS neurons labeled by DTMR (2nd TX). Scale, 100 μm. **(D)** At 1 month post-TX, RS neurons transfection efficiency between MO_Ctrl_ (*n* = 9) and ${\text{MO}}_{{\text{PTP}}_{\text{σ}}}$ (*n* = 8) are comparable, approximately 72–80% of identified neurons. However, at 6 months post-TX, MO_Ctrl_ persists in 70% of RS neurons while MO is present in only 32%. The analysis was performed with two-way ANOVA with a *post hoc* Sidak test. Error bars show SEM. ***p < 0.001. **(E)** Pearson’s correlation analysis revealed a strong positive correlation between labeling from the 1st and 2nd TX at 6 months. Thus, the reduction in ${\text{MO}}_{{\text{PTP}}_{\text{σ}}}$ labeling is from genuine cell loss and not degradation of the MO. **p < 0.01; n.s., non-significant.

**Figure 4:**
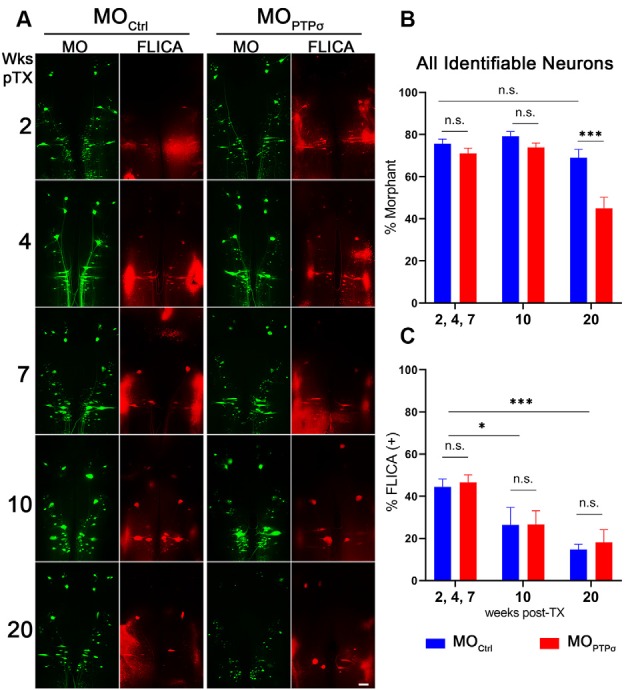
PTPσ knockdown-mediated reduction in neuronal survival was not accompanied by enhanced caspase activation. **(A)** Representative whole mounted brain widefield fluorescence micrographs of RS neurons labeled with MO_Ctrl_ or ${\text{MO}}_{{\text{PTP}}_{\text{σ}}}$ (green) and poly caspase FLICA (red) after TX. Scale bar, 200 μm. Quantification of MO labeling **(B)** and MO+FLICA double-labeling **(C)** among all identified RS neurons (*n* = 5–9 lampreys per group per time point). Despite exacerbated cell loss, enhanced caspase activation was not detected following TX and PTPσ KD. Statistical analysis performed with two-way ANOVA and Sidak *post hoc* multiple comparisons test. Error bars show SEM. *p < 0.05; ***p < 0.001; n.s., non-significant.

In these studies, cell survival was assessed using labeling by fluorescently-tagged MOs. By design, MOs are persistent and extremely resistant to degradation by endonucleases. At 1 month post-TX, both control and PTPσ MOs transfected the identified RS neurons with comparable efficiency (Figure 3). To confirm whether MO labeling persisted over long time periods, a small cohort of lampreys administered ${\text{MO}}_{{\text{PTP}}_{\text{σ}}}$ or MO_Ctrl_ were subjected to a second TX, 6 months after the first, and back labeled with DTMR. Importantly, this TX was made *rostral* to the first, at the level of the third gill, so as to label surviving identified RS neurons. The analysis revealed that the MO fluorescence almost perfectly co-labeled with the dextran dye. Quantitatively, this manifested as a strong positive correlation between the numbers of neurons labeled during each TX (*r* = 0.97, *n* = 5 lampreys total). This confirmed that the MO labeling persists even at extended time points, supporting the use of MO labeling as a proxy for cell survival. Since transfection efficiency was comparable between the control- and PTPσ-targeting MOs at 1-month post-TX, loss of MO-labeling at extended timepoints reflected genuine cell loss, and was not a result of differences in labeling efficiency or stability.

### PTPσ Knockdown Did Not Enhance TX-Induced Caspase Activation

In lampreys, TX induces a delayed form of apoptotic retrograde death among “bad regenerator” identified RS neurons (Shifman et al., 2008). Because the reduction in cell survival observed among ${\text{MO}}_{{\text{PTP}}_{\text{σ}}}$ lampreys was highly unexpected, we assessed the post-TX time course of apoptosis signaling to determine if the observed cell loss was due to enhanced caspase activity. After PTPσ knockdown, we processed wholemount lamprey brains for activated caspases with poly caspase FLICA at 2, 4, 7, 10 and 20 weeks post-TX (Figure 4). In this cohort, significant identified RS neuron loss was seen in MO animals at 20 weeks post-TX, but not at earlier time points. Surprisingly, this cell loss was not preceded by enhanced caspase activation.

### PTPσ Knockdown Did Not Enhance Akt Phosphorylation or p53 Abundance

**Figure 5:**
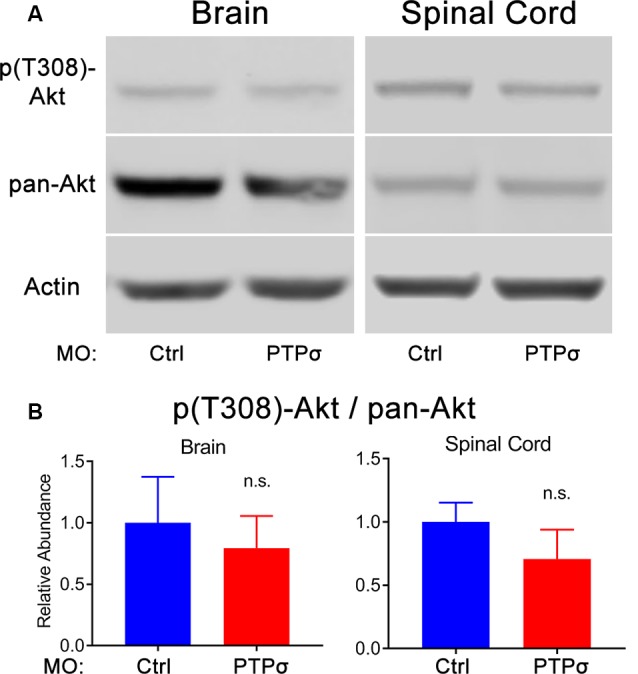
PTPσ KD did not promote Akt phosphorylation. **(A)** Representative Western Blot images and quantification for phospho-Akt, pan-Akt, and Actin in lamprey brain homogenates (*n* = 5) or spinal cord (*n* = 4–5) 2 weeks post-TX. **(B)** Analysis of relative signal intensities was performed with two-tailed student’s *t*-test (*p* > 0.05 for all comparisons). Error bars show SEM; n.s., non-significant.

The molecular intermediaries in the LAR-family RPTP signaling pathway are still not completely known. The primary downstream effect of CSPG-PTPσ signaling is believed to be activation of the small GTPase, RhoA, which regulates cytoskeletal dynamics *via* its associated kinase, ROCK (Ohtake et al., 2016). An additional, but important consequence of CSPG-induced LAR-family RPTP activation is inhibition of the pro-survival, pro-growth Akt pathway, likely *via* ROCK but potentially through RhoA-independent mechanisms. This second effect may be particularly important because, at least in lampreys, axon regeneration in the CNS appears to be accomplished in the absence of F-actin-filled growth cones (Lurie et al., 1994). Moreover, since Akt regulates cell survival following PTPσ knockdown, its activity is especially relevant, given the observed loss of axotomized RS neurons following PTPσ knockdown. To assess Akt activity, Western blots were performed on lamprey brains and spinal cords. Contrary to our original hypothesis, but consistent with RS neuronal loss, we did not observe increases in phospho-Akt (Thr308) levels among ${\text{MO}}_{{\text{PTP}}_{\text{σ}}}$-treated lampreys, 2 weeks post-TX, in both brains and spinal cords extending from approximately the second gill to 0.5 cm below the TX. If anything, in both the brain and spinal cord, the relative abundance of activated Akt was reduced, though this did not reach statistical significance (Figure 5). Additionally, total Akt levels were unchanged relative to controls.

**Figure 6:**
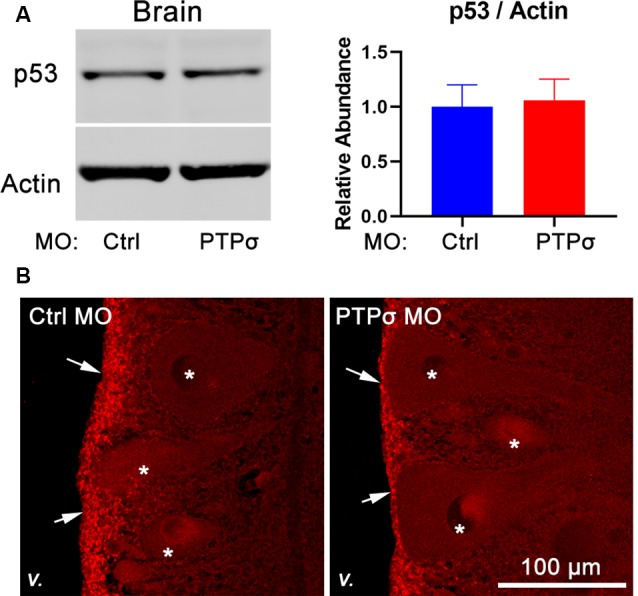
${\text{MO}}_{{\text{PTP}}_{\text{σ}}}$ transfection did not promote p53 activation. **(A)** Representative Western Blot image and quantification for p53 and actin in lamprey brain homogenates (*n* = 4–5). **(B)** Representative confocal maximum intensity projections of p53 immunofluorescence in lamprey hindbrain sections (*v*., 4th ventricle; arrows, ependyma; *reticulospinal neurons).

Given the unexpectedness of PTPσ knockdown-associated cell death, it was important to consider off-target effects. In zebrafish, off-target MO toxicity typically is p53-dependent (Bedell et al., 2011; Gerety and Wilkinson, 2011). Although p53 is complexly regulated, a major consequence of activation is a several-fold increase in protein half-life (Giaccia and Kastan, 1998). Thus, to assess p53 activation in our model, we assessed p53 abundance with Western Blot and immunofluorescence 2 weeks after TX and MO transfection (Figure 6). Overall, p53 abundance in lamprey brains was low, with expression primarily restricted to the ependymal cells lining the ventricles. No appreciable differences were observed between animals treated with the control or PTPσ-targeting MO. Likewise, Western Blot analysis showed similar levels of p53 expression between both groups of brain homogenates. Although these results cannot conclusively rule out all potential off-target effects, they do exclude the most common pathway for cell-autonomous MO toxicity. Additionally, the long delay between confirmed MO biological activity in this study (i.e., within 2 weeks post-TX) and neuron loss (10–20 weeks post-TX) seems uncharacteristic of acute toxicity. Finally, it is important to note, that MOs have been used repeatedly in lamprey studies, and, to date, no off-target effects have been reported (Zhang et al., 2015; Hu et al., 2017; Sobrido-Cameán et al., 2019).

### PTPσ Knockdown Attenuated the Local Immune Response to TX

**Figure 7:**
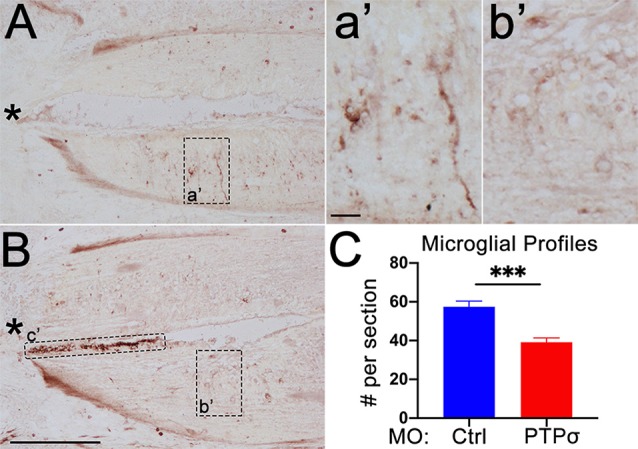
PTPσ knockdown attenuated the local immune response to TX. **(A,B)** Representative micrographs of IB4 staining in rostral horizontal spinal cord sections from MO_Ctrl_ **(A,a’)** and MOPTPσ **(B,b’)** lampreys 2 weeks pTX. Infiltrating macrophages in the central canal **(c’)** were excluded from the analysis. **(C)** On mean, fewer IB4+ microglia were present in sections from MO spinal cords (*n* = 2 animals, eight sections) than MO_Ctrl_ spinal cords (*n* = 3 animals, 12 sections). Analysis was performed with a two-tailed student’s *t*-test. Error bars show SEM. ***p < 0.001. Scale, 200 μm **(A,B)**, 20 μm** (a’,b’)**. *TX.

Although MO transfection in the brain was limited to those RS neurons whose axons extended to at least the 5th gill, it was possible that PTPσ-expressing cells adjacent to the TX, including resident microglia and infiltrating macrophages, were incidentally transfected. As perturbations to the inflammatory response could contribute to the RS cell loss observed after PTPσ knockdown, we assessed the number of microglial profiles present in horizontal spinal cord sections, extending 600 μm rostral of the TX, 2 weeks after injury (Figure 7). Unexpectedly, we observed a significant reduction in the number of microglial profiles in the spinal cords of lampreys treated with ${\text{MO}}_{{\text{PTP}}_{\text{σ}}}$. This suggests that PTPσ knockdown may have attenuated the inflammatory response to SCI.

### PTPσ Knockdown Modestly Impaired Behavioral Recovery From TX

In lampreys, behavioral recovery of coordinated swimming after TX is mediated by true axon regeneration (Selzer, 1978). However, the role of the identified RS neurons in restoring locomotion is less clear. Although under certain conditions, stimulation of these neurons may elicit movement, partial injury models suggest that they are not necessary to initiate locomotion (Rovainen, 1967; McClellan, 1988). Thus, it is not currently possible to associate behavioral recovery with anatomical regeneration of specific neurons. Nevertheless, swimming recovery after TX is a useful correlate for regenerative and plastic processes in toto, as well as a reflection of overall animal health.

**Figure 8:**
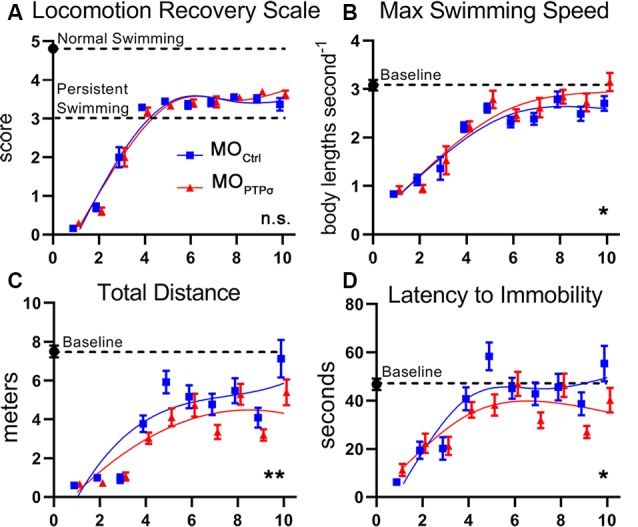
PTPσ knockdown modestly impaired behavioral recovery from TX. **(A)** Semiquantitative movement recovery score: see text. Immediately after TX, lampreys were paralyzed below the injury but over the course of 4 weeks recovery coordinated swimming. However, postural deficits persisted beyond 10 weeks of injury. No differences in swimming quality were observed between MO-Ctrl and MO-PTPσ treated lampreys. **(B–D)** Quantitative swimming parameters assessed by AnyMaze animal tracking software during 2 min of open field swim. Deficits are observed after TX but slowly trend towards baseline over the course of 10 weeks. PTPσ KD modestly reduced recovery on Total Distance Traveled, and Latency to Immobility. However, PTPσ KD slightly outperformed controls on maximum swimming speed. Analysis was performed with two-way ANOVA. Error bars show SEM. *p < 0.05; **p < 0.01; n.s., non-significant.

One of the more common approaches to assess swimming recovery is the use of semiquantitative locomotion scales, which vary slightly from study-to-study (Ayers, 1988; Herman et al., 2018). In the present report, locomotion quality was assessed during 2 min of free swim recorded 3 days per week, 1 week before the injury and up to 10 weeks after TX, using a locomotion recovery scale, which assessed qualitative aspects of lamprey swimming on a five point scale (Figure 8). Uninjured lampreys are efficient swimmers and consistently maintained an upright dorsal-ventral posture (score 5). After injury, lampreys were paralyzed below the injury (score 0) but, during the first few weeks of recovery, experienced tonic body flexion movements below the lesion (score 1) and gained the ability to propel themselves forward short distances (stage 2). By 4 weeks post-injury, lampreys recovered the ability to swim but are unable to maintain proper dorsal-ventral posture (scores 3 and 4). Although dorsal-ventral posture improved gradually, swimming remained abnormal for at least 10 weeks post-TX. No differences in swimming quality were observed between MO_Ctrl_ and ${\text{MO}}_{{\text{PTP}}_{\text{σ}}}$ lampreys.

To increase the sensitivity of our swimming assay, we used ANY-maze software (Stoelting) to quantify kinematic parameters including maximum speed, total distance traveled, and latency to immobility. Notably, our observation of the maximum swimming speed of approximately three body lengths seconds^−1^ in uninjured lampreys was consistent with previous reports (Gemmell et al., 2016). As with the semiquantitative assessment, deficits were clearly observed immediately following TX but improved over the following weeks. Interestingly, during late recovery, maximum speed approximated control levels despite persistent deficits in the righting response. Total distance traveled remained minimal after TX until 4 weeks when swimming ability returned but continued to remain below baseline for the duration of the study. Latency to immobility showed deficits before 4 weeks but afterward approximated baseline. Although modest deficits were observed in total swim distance and latency to immobility, it is clear that PTPσ knockdown did not grossly alter behavioral recovery following TX.

## Discussion

Contrary to our original hypothesis, knocking down lamprey PTPσ with antisense MOs reduced, rather than improved, RS axon regeneration and cell survival after TX. Indeed, beginning at 10 weeks post-TX, PTPσ knockdown significantly reduced survival of the identified RS neurons. This delay in effect is comparable to the long delay in TX-induced apoptosis previously reported among the poorly-regenerating RS neurons (Shifman et al., 2008). Typically, this injury-induced supraspinal cell death is preceded by activation of caspases, which can be detected within cell bodies as early as 2 weeks post-TX (Barreiro-Iglesias and Shifman, 2012; Hu et al., 2013). However, in the present study, exacerbated cell death following PTPσ knockdown was not accompanied by a similar increase in the activation of caspases. Although effects on swimming recovery were modest, the changes were in the same direction as the reduction in cell survival and axon regeneration. PTPσ knockdown did not induce significant changes in Akt phosphorylation in the brain or spinal cord lysates.

Although this study did not identify the mechanism by which PTPσ knockdown enhanced TX-induced cell death, the findings indicate that PTPσ does not significantly suppress RS axon regeneration in lampreys. This interpretation runs contrary to the putative role of PTPσ as an important receptor mediating the axon growth-inhibiting effects of CSPGs in mammalian SCI. However, there are a number of on-target cell-autonomous and non-autonomous mechanisms that may explain our findings, as discussed below.

### PTPσ Interactions With CSPGs and HSPGs

Like SCI in mammals, lamprey TX results in “scar” formation around the lesion. In mammals, the scar is characterized by a glia limitans of reactive astrocytes surrounding a fibrotic core (Wanner et al., 2013). In lampreys, the primary feature of the lamprey scar is an enlarged central canal and realignment of glial processes from a transverse to longitudinal alignment, which bridges proximal and distal spinal stumps (Yin and Selzer, 1983; Lurie et al., 1994). In both cases, the scar is rich in growth-inhibiting CSPGs (Lander et al., 1998; McKeon et al., 1999; Stichel et al., 1999; Thon et al., 2000; Jones et al., 2002, 2003; Zhang et al., 2014). Receptors on axon tips, including the LAR-family RPTPs, PTPσ, and LAR; as well as the myelin-associated inhibitor receptors, NgR1 and NgR3, are believed interact with extracellular CSPGs to induce growth cone collapse, halting axon outgrowth (Shen et al., 2009; Fisher et al., 2011; Dickendesher et al., 2012). PTPσ and LAR generally colocalize among lamprey bad regenerator RS neurons (Zhang et al., 2014; Rodemer et al., 2015). Lamprey RS neurons also express an NgR homolog, although its distribution has yet to be well characterized (unpublished observations). Thus, it possible that any regeneration promoting effects of PTPσ KD was compensated for by other CSPG receptors.

Alternatively, HSPGs compete with CSPGs for PTPσ binding. Unlike growth-inhibitory CSPGs, HSPGs—PTPσ interactions *promote* neurite outgrowth, although the mechanism remains unclear. *In vitro* studies suggest PTPσ—HSPG interactions stabilize the receptor in tight clusters on the plasma membrane resulting in uneven distribution of intracellular phosphatase activity (Coles et al., 2011). However, other studies suggest both CSPGs and HSPGs induce clustering but that HSPGs interact with PTPσ *via* a secondary binding site (Katagiri et al., 2018). Regardless of the exact mechanism, HSPGs—PTPσ interactions may predominate the lamprey spinal cord resulting in PTPσ having a net pro-growth, pro-survival role after TX. Moreover, the sulphation patterns on the CSPG GAG side chains are believed to greatly influence receptor interactions. Notably, PTPσ has been shown to preferentially interact with di-sulfated CSPGs and only weakly interact with mono-sulfated CSPGs, including CS-C, the dominant CSPG species in the glial scar (Katagiri et al., 2018; Tadai et al., 2018). While CSPGs in the mammalian glial scar may contain sufficient GAG diversity to preserve PTPσ binding, CSPGs in lampreys remain largely uncharacterized. Thus, depending on sulphation patterns, it is possible that lamprey PTPσ does not strongly interact with the CSPG scar.

### The Role of PTPσ in Non-neuronal Cells

Although most studies on PTPσ have focused on neuron-intrinsic effects, PTPσ is also expressed in non-neuronal cells. Reports have suggested that CSPGs antagonize neural progenitor and oligodendrocyte precursor cell survival, proliferation, and differentiation, which can be rescued by deleting PTPσ or inhibiting receptor function with peptides (Dyck et al., 2015, 2019; Zhong et al., 2019). Notably, PTPσ is also expressed within the immune system among subpopulations of dendritic cells and, in lower levels among T-cells and microglia (Bunin et al., 2015; Ohtake et al., 2017). While the lamprey immune system is unique, it includes analogs to mammalian immune components including B- and T-like cells, and microglia (Laramore et al., 2011; Hirano et al., 2013). In this study, MO-transfection in the brain was clearly limited to neurons with projections extending at least to the 5th gill. However, it was possible that the application of MO to the spinal cord stump incidentally transfected local populations, reducing PTPσ expression on those cells and, consequently, altering their response to TX. Using isolectin B4 staining, we observed a significant reduction in the number of microglial profiles present in the spinal cord at, and adjacent to the TX in PTPσ knockdown lampreys, which suggests a possible attenuated immune response to injury. However, our approach did not distinguish small microglial cell bodies from large, transversely sectioned microglial processes, so the apparent reduction in microglia number might be due to differences in the activation state of microglia, rather than to reductions in microglial proliferation or infiltration. Moreover, the question of whether this perturbation to the immune response is beneficial or detrimental to neuron survival and regeneration is not yet determined.

Interestingly, there are conflicting reports in mammals as to whether PTPσ inhibition reduces or promotes inflammation. In a mouse MOG-induced model of experimental allergic encephalomyelitis (EAE), genetic deletion of PTPσ exacerbated inflammation within the spinal cord, resulting in increased infiltration of CD4+ T-cells, activation of macrophages, and upregulation of the pro-inflammatory cytokines, which led to enhanced axon damage and worsened clinical outcome (Ohtake et al., 2017). However, in another EAE report, which utilized intraperitoneal injection of blocking peptides, PTPσ inhibition reduced inflammation and improved clinical EAE scores (Luo et al., 2018). In a rat clip-compression model of SCI, PTPσ blocking peptides shifted immune response from pro- to anti-inflammatory, including upregulation of anti-inflammatory cytokines and increased activation of “M2” microglia and T-regulatory cells (Dyck et al., 2018). In that study, while there was a shift in the microglial activation state towards M2, no change was observed in total microglia number. In the present study, although the exact significance of the reduction in microglial density associated with PTPσ knockdown has not yet been established, the findings suggest that non-autonomous processes at the site of injury may have contributed to impaired supraspinal RS neuron survival following PTPσ knockdown.

### Alternative Cell-Autonomous Effects

During neural development, LAR-family RPTPs, including PTPσ, function as important organizers of excitatory synaptogenesis (Han et al., 2018; Südhof, 2018; Bomkamp et al., 2019). *In vitro* and biochemical studies suggest that presynaptic PTPσ forms a trans-synaptic adhesion complex with postsynaptic proteins *via* its Ig-like and fibronectin-type III extracellular domains. These postsynaptic proteins include NGL-3, TrkC, IL1RAPL1, IL1RAcP, Slitrk 1-6, and SALM-3 (Takahashi et al., 2011; Yoshida et al., 2011, 2012; Um et al., 2014; Li et al., 2015). Although studies with primary neuronal cultures have shown that PTPσ impairs synapse formation *in vitro*, evidence has been lacking *in vivo* (Han et al., 2018). Notably, in a recent study using an AP-tagged fusion construct in mouse brain tissue slices, PTPσ interacted directly with neurons, and binding persisted even after CSPGs and HSPGs were enzymatically digested (Yi et al., 2014). Although it remains unclear whether PTPσ deficiency impairs regenerative synaptogenesis *in vivo*, it is possible that this may have contributed to the neuron loss observed in our model *via* trophic deprivation.

Alternatively, an RNAi screen in U2OS cells has implicated PTPσ as a negative regulator of autophagy (Martin et al., 2011). In these cells, PTPσ inhibition hyperactivated both constitutive and induced autophagic pathways by relieving downregulation of phosphatidylinositol-3-phosphate (PtdIns3P), a critical lipid involved in the formation of autophagic vesicles. Although cell death was not reported in that study, it is possible that prolonged PTPσ deficiency promotes aberrant hyperactivation of autophagy pathways *in vivo*. Thus, non-apoptotic, autophagy-dependent mechanisms may have driven cell death following PTPσ knockdown in our model (Denton and Kumar, 2019). Obviously, further investigations will be needed to test these hypotheses.

## Conclusion

The results from this study suggest that PTPσ is not a net negative regulator of axon regeneration in the RS neurons of lamprey. Although this result is not consistent with the putative role of PTPσ in mammal axon regeneration, it can potentially be explained by known cell-autonomous or non-autonomous mechanisms. In this report, PTPσ knockdown significantly reduced regeneration and antagonized RS neuron survival beginning between 10 and 20 weeks post-TX. This cell loss was not accompanied by enhanced caspase activation. To our knowledge, PTPσ deletion has not been previously implicated in neuron death; nor, however, has it been shown to be neuroprotective. For example, following optic nerve crush, PTPσ deletion reportedly improved axon regeneration but did not prevent retinal ganglion cell death (Sapieha et al., 2005). The lack of activated caspases and long latency suggest the possibility that enhanced supraspinal neuron death following MO-induced PTPσ knockdown resulted from non-apoptotic mechanisms.

## Data Availability Statement

The datasets generated for this study are available on request to the corresponding author.

## Ethics Statement

The animal study was reviewed and approved by Temple University’s Institutional Animal Care and Use Committee.

## Author Contributions

WR designed, performed, and analyzed the experiments, and wrote the first draft of the manuscript. GZ designed and performed experiments. IS and LJ performed experiments. JH performed experiments and contributed to the writing of the manuscript. SL contributed to the experimental design and suggested specific experiments. MS designed experiments and contributed to writing the manuscript.

## Conflict of Interest

The authors declare that the research was conducted in the absence of any commercial or financial relationships that could be construed as a potential conflict of interest.
